# Determination of 60 Migrant Substances in Plastic Food Contact Materials by Vortex-Assisted Liquid-Liquid Extraction and GC-Q-Orbitrap HRMS

**DOI:** 10.3390/molecules26247640

**Published:** 2021-12-16

**Authors:** Pablo Miralles, Vicent Yusà, Yovana Sanchís, Clara Coscollà

**Affiliations:** 1 Foundation for the Promotion of Health and Biomedical Research in the Valencian Region (FISABIO), Avinguda Catalunya 21, 46020 Valencia, Spain; miralles_pabiba@gva.es (P.M.); yusa_vic@gva.es (V.Y.); sanchis_yov@gva.es (Y.S.); 2 Department of Analytical Chemistry, University of Valencia, Avinguda Doctor Moliner 50, 46100 Burjassot, Spain; 3 Public Health Laboratory of Valencia, Avinguda Catalunya 21, 46020 Valencia, Spain

**Keywords:** food contact materials, food simulants, gas chromatography, high-resolution mass spectrometry, migration test, target analysis

## Abstract

A GC-HRMS analytical method for the determination of 60 migrant substances, including aldehydes, ketones, phthalates and other plasticizers, phenol derivatives, acrylates, and methacrylates, in plastic food contact materials (FCM) has been developed and validated. The proposed method includes migration tests, according to Commission Regulation (EU) 10/2011, using four food simulants (A, B, C, and D1), followed by vortex-assisted liquid–liquid extraction (VA-LLE) and GC-Q-Orbitrap HRMS analysis in selected ion monitoring (SIM) mode, with a resolving power of 30,000 FWHM and a mass accuracy ≤5 ppm. The method was validated, showing satisfactory linearity (R^2^ ≥ 0.98 from 40 to 400 µg L^−1^), limits of quantification (40 µg L^−1^), precision (RSD, 0.6–12.6%), and relative recovery (81–120%). The proposed method was applied to the analysis of field samples, including an epoxy-coated tin food can, a drinking bottle made of Tritan copolyester, a disposable glass made of polycarbonate, and a baby feeding bottle made of polypropylene, showing that they were in compliance with the current European regulation regarding the studied substances.

## 1. Introduction

In Europe, the ‘Commission Regulation (EU) No 10/2011 of 14 January 2011 on plastic materials and articles intended to come into contact with food’ [1] (henceforth, Regulation 10/2011) contains the ‘Union list of authorized substances‘ (IAS), which establishes the specific migration limits (SML) that they should comply with in order to ensure the safety of the consumers. Moreover, non-intentionally added substances (NIAS) could also be present in plastic food contact materials (FCM) as impurities of the authorized substances, reaction, or degradation products, formed during the manufacture, storage, or consumption processes [2]. In this sense, the development and validation of analytical methods for the quantification of migrant substances, both IAS and NIAS, in plastic FCM using food simulants are a matter of importance and a research field of growing interest [3,4,5,6].

In the analytical literature, several works regarding the analysis of migrant substances from plastic FCM have been published in recent years. Some of them use screening or non-target approaches for the identification of IAS, NIAS, and unknown migrant substances [7,8,9,10,11,12,13,14], while other published papers deal with the target analysis of selected migrant substances, such as phthalates and plasticizers by gas chromatography (GC) coupled to mass spectrometry (MS) [15,16,17,18]; light stabilizers and antioxidants by liquid chromatography (LC) coupled to ultraviolet-visible spectrophotometry (UV) [19,20], tandem mass spectrometry (MS/MS) [21], and high-resolution mass spectrometry (HRMS) [22]; phenolic compounds, such as 4-nonylphenol by GC-MS [23]; bisphenols, their diglycidyl ethers, and other derivatives by GC-MS [24], LC-UV [25], and LC-HRMS [26]; and primary aromatic amines by GC-MS [27], LC-MS/MS [28], and LC-HRMS [29], among other groups of substances. More information regarding the analytical methods for the target analysis of migrant substances from plastic FCM can be found in several recent review articles published on this topic [4,5,30,31,32,33].

While most of the published studies are focused on the analysis of particular families or groups of substances with a reduced number of target analytes, few multi-residue or multi-analyte methods are available in the analytical literature. The most significant are the determination of 48 contaminant residues in food contact plastic products by microwave-assisted extraction, using methanol as an alternative ‘non-regulated’ food simulant, followed by LC-HRMS, published by Zhang et al. [34]; the determination of 84 migrant substances using food simulants A and C, followed by liquid–liquid extraction using dichloromethane as extraction solvent and GC-MS, published by Tsochatzis et al. [35]; and the determination of 75 migrant substances using food simulants A, C, and D1, followed by salt-assisted liquid–liquid extraction using dichloromethane as extraction solvent and GC-MS/MS, also published by Tsochatzis et al. [36]. Moreover, GC presents the handicap of not being directly compatible with the hydro-ethanolic food simulants of Regulation 10/2011 [1]. For this reason, it is required to use alternative food simulants [17,18,23], such as organic solvents, or to perform a liquid-phase [15,35,36] or a solid-phase extraction [24,27] in order to enable the GC analysis. With regard to the detection technique, currently there are no published methods that use GC coupled to HRMS, which enhances the identification and confirmation confidence of the migrant substances by means of their exact masses.

To the best of our knowledge, this is the first work addressing a simple and reliable analytical procedure for the determination of 60 migrant substances in plastic FCM using four food simulants (A, B, C, and D1), according to Regulation 10/2011 [1], followed by vortex-assisted liquid–liquid extraction (VA-LLE), using only n-hexane as an extraction solvent, and GC-Q-Orbitrap HRMS analysis.

However, in the current market, there is a wide variety of FCM made of different plastics and polymer materials, such as epoxy-coatings, polypropylene (PP), polyethylene (PE), polyethylene terephthalate (PET), polycarbonate (PC), and polyester copolymers, among others. Furthermore, not all the studied substances, both IAS and NIAS, may be relevant for all the plastic FCM. In this sense, a fast and reliable multi-analyte method to quantify a large set of GC-suitable migrant substances, such as those proposed in the present manuscript, could be useful for food control authorities in order to evaluate compliance of plastic FCM with current European regulations as a part of a comprehensive analytical control plan [1,37].

## 2. Results and Discussion

### 2.1. Selection of the Target Analytes

The abovementioned Regulation 10/2011 [1], in its ‘Union list of authorized substances’, contains approximately one thousand IAS and their applicable restrictions, including SML. Moreover, NIAS should also be controlled to ensure that their migration does not surpass the established limits [1].

From this enormous number of substances to be controlled, the selection of the target analytes was performed on the basis of two criteria: their analytical standards should be commercially available, and they should be suitable for determination with the selected analytical technique, GC-HRMS (mainly hydrophobic organic compounds). In this sense, inorganic substances, such as oxides, hydroxides, silicates, and metals; macromolecules and oligomers, such as sugars, waxes, and resins; and highly polar compounds, such as acids, alcohols, and amines were not considered. Finally, a representative set of 60 substances, including aldehydes, ketones, phthalates and other plasticizers, phenol derivatives, acrylates, and methacrylates, among others, were selected as target analytes (see Table 1). However, this list of target analytes was not meant to be exhaustive, and it could be extended if necessary.

### 2.2. Selection of the Quantification and Confirmation Ions

In order to select the quantification and confirmation ions of the target analytes for their acquisition in SIM mode (see Section 3.4.4), individual standard solutions containing the target analytes at 400 µg L^−1^ in n-hexane were prepared and injected directly into the GC-HRMS system in full scan mode with a resolving power of 60,000 FWHM, in order to obtain their HRMS spectrum. For each target substance, the most abundant fragment ions were selected as quantification and confirmation ions, respectively (see Table S1). If possible, the molecular formulas of the selected fragment ions were obtained, and their theoretical exact masses were considered for further acquisition.

**Figure 1:**
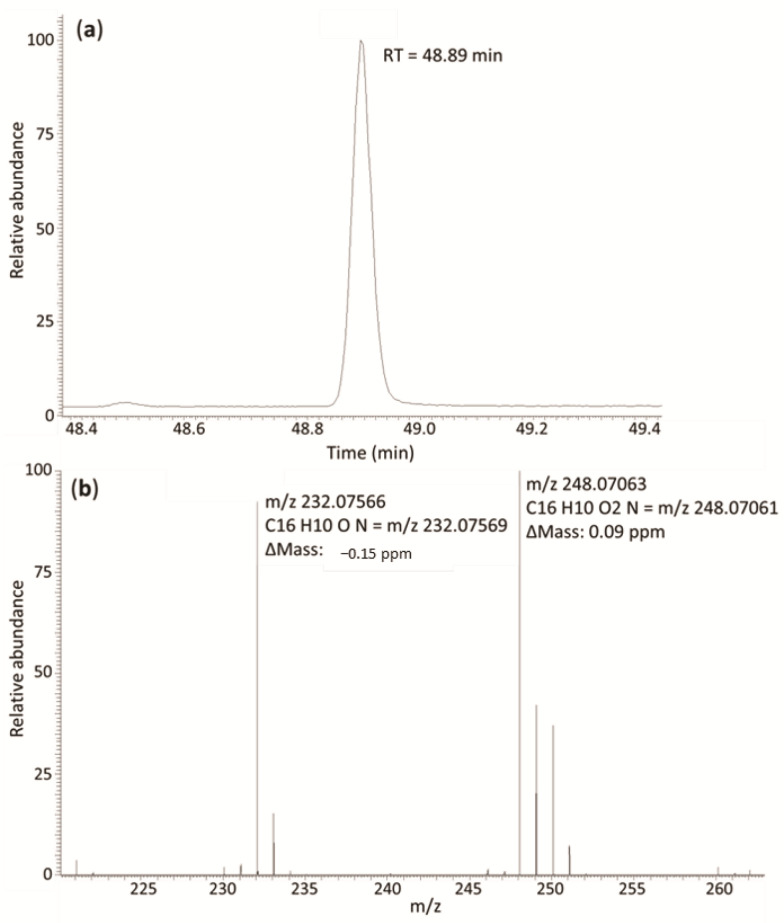
Acquired features for Octoctylene: (**a**) Chromatographic peak; (**b**) HRMS spectrum with the exact masses of selected fragment ions.

As an example, the chromatographic peak and the selected fragment ions for Octocrylene, with their exact masses, are shown in Figure 1.

### 2.3. Study of the Experimental Variables Involved in the VA-LLE Procedure

In order to make the aqueous (or hydro-ethanolic) food simulants obtained from the migration test of the samples (see Section 3.4.1) compatible with the selected analytical technique (GC-HRMS), VA-LLE with an organic solvent was performed.

The experimental variables involved in the extraction process were studied, in triplicate, and those which provided the highest analytical signal (peak area) for a broad number of target compounds were selected. In this sense, extraction solvent composition and volume, ionic strength, and extraction time were studied. To perform these studies, a standard solution containing the target analytes at 160 µg L^−1^ in food simulant A was used. The volume of the food simulant was fixed at 20 mL.

#### 2.3.1. Extraction Solvent Composition

**Figure 2:**
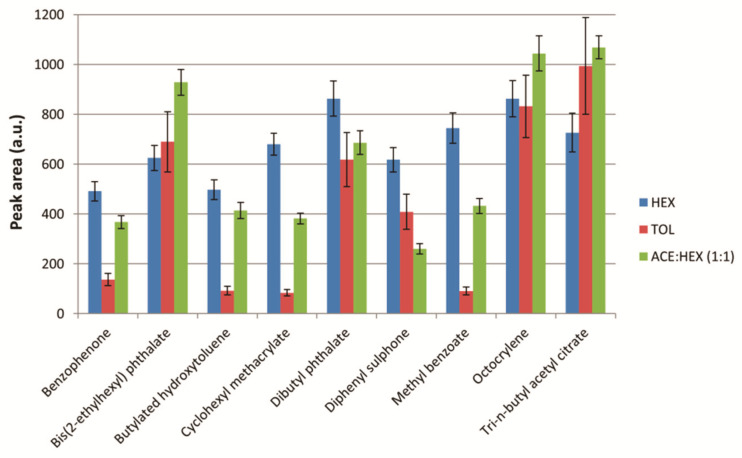
Study of the extraction solvent composition. Experimental conditions: extraction solvent volume, 5 mL; ionic strength, 5% *w*/*v* of NaCl; extraction time, 60 s. Bars in the graph represent the mean value of three replicates ± standard deviation. HEX: n-hexane; TOL: toluene; ACE:HEX: mixture of acetone/n-hexane (1:1).

The organic solvent to perform the extraction had to be immiscible and less dense than water in order to facilitate the collection of the extract as a supernatant phase. In this sense, n-hexane, toluene, and a mixture of acetone/n-hexane (1:1) were tested as extraction solvents. Moreover, chlorinated solvents were not considered due to their higher density and toxicity. The conditions for the other variables were as follows: extraction solvent volume, 5 mL; ionic strength, 5% *w*/*v* of NaCl; extraction time, 60 s. The obtained results are shown in Figure 2. It should be noted that only a representative set of nine target analytes (benzophenone, bis(2-ethylhexyl) phthalate, butylated hydroxytoluene, cyclohexyl methacrylate, dibutyl phthalate, diphenyl sulphone, methyl benzoate, octocrylene, and tri-n-butyl acetyl citrate) have been included in the figures below in order to simplify the visualization of the results. These substances were selected for being the most common and/or representative of the different chemical families studied in the present manuscript. Similar trends and conclusions were observed for the other target compounds.

As can be seen, the best results were obtained with n-hexane, so it was selected for further analysis.

#### 2.3.2. Extraction Solvent Volume

**Figure 3:**
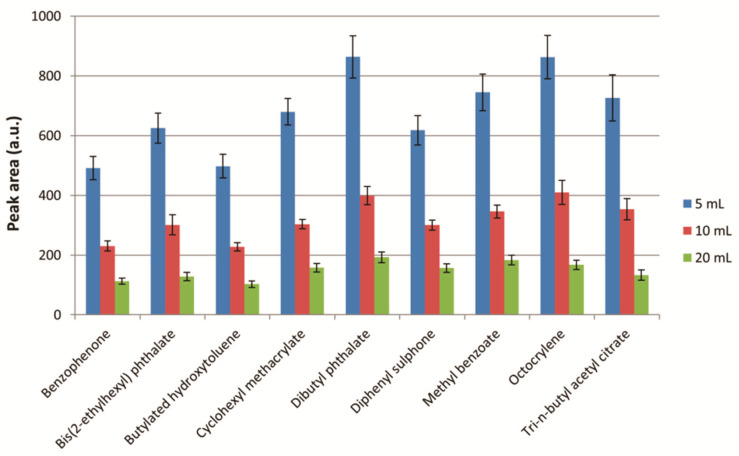
Study of the extraction solvent volume. Experimental conditions: extraction solvent, n-hexane; ionic strength, 5% *w*/*v* of NaCl; extraction time, 60 s. Bars in the graph represent the mean value of three replicates ± standard deviation.

The mass transfer of the analytes from the food simulant to the extraction solvent depends on the ratio between the food simulant and the extraction solvent volumes. In this sense, the volume of the food simulant was fixed at 20 mL, and 5, 10, and 20 mL were tested as extraction solvent volumes. The conditions for the other variables were as follows: extraction solvent, n-hexane; ionic strength, 5% *w*/*v* of NaCl; extraction time, 60 s. The obtained results are shown in Figure 3.

From the point of view of the mass transfer of the analytes, a higher volume of extraction solvent implies that a higher amount of the analytes could be extracted, but the obtained extract would be more diluted. As can be seen, the analytical signal decreased with higher volumes due to the dilution effect, so 5 mL of extraction solvent were selected for further analysis. Moreover, the obtained sensitivity using the selected volume of extraction solvent allowed us to reduce the organic solvent consumption of the proposed method and to avoid further experimental steps, such as repeated extractions or evaporation and reconstitution steps.

#### 2.3.3. Ionic Strength

**Figure 4:**
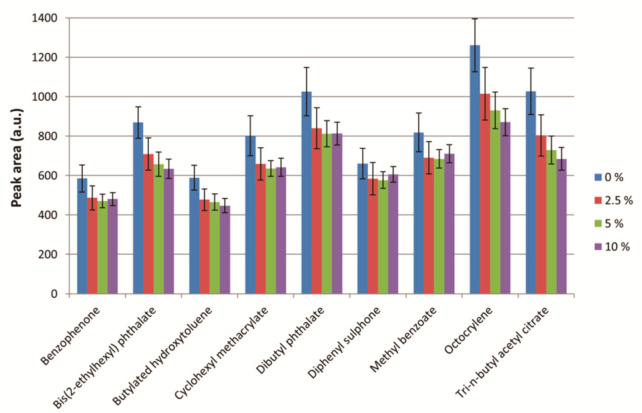
Study of the ionic strength. Experimental conditions: extraction solvent, n-hexane; extraction solvent volume, 5 mL; extraction time, 60 s. Bars in the graph represent the mean value of three replicates ± standard deviation.

The influence of the ionic strength of the food simulant was studied in order to evaluate the so-called ‘salting-out effect’. For this, additions of 0, 2.5, 5, and 10% *w*/*v* of NaCl were tested. The conditions for the other variables were as follows: extraction solvent, n-hexane; extraction solvent volume, 5 mL; extraction time, 60 s. These results are shown in Figure 4.

As can be seen, the best results were obtained with the addition of 0% *w*/*v* of NaCl, so no adjustment of the ionic strength was selected for further analysis.

#### 2.3.4. Extraction Time

**Figure 5:**
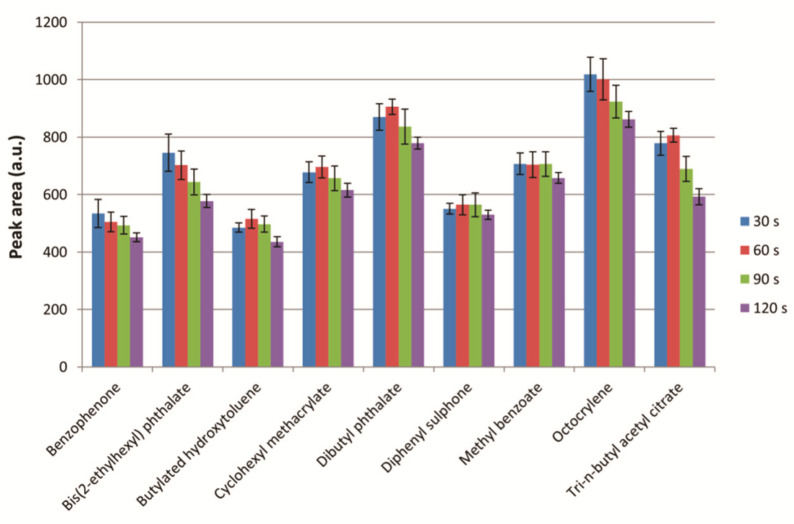
Study of the extraction solvent volume. Experimental conditions: extraction solvent, n-hexane; extraction solvent volume, 5 mL; ionic strength, not adjusted. Bars in the graph represent the mean value of three replicates ± standard deviation.

The influence of the extraction time, as the time of vortex mixing, was studied. For this, 30, 60, 90, and 120 s were tested. The conditions for the other variables were as follows: extraction solvent, n-hexane; extraction solvent volume, 5 mL; ionic strength, not adjusted. These results are shown in Figure 5.

As can be seen, similar results were observed between 30 and 60 s, so the shortest extraction time of 30 s was selected for further analysis. With 90 and 120 s, a decrease in the analytical response was observed for some of the target analytes. This may be due to kinetic or thermodynamic effects that alter the partition coefficient of the analytes between the food simulant and the extraction solvent with longer extraction times.

### 2.4. Study of the Extraction Efficiency

In order to evaluate the extraction efficiency of the proposed VA-LLE under the selected conditions, a standard solution containing the target analytes at 160 µg L^−1^ in n-hexane was directly injected into the GC-HRMS system, and the obtained analytical signal was compared with that obtained for a standard solution containing the target analytes at 160 µg L^−1^ in food simulant after performing the extraction process (see Section 3.4.3).

Considering the ratio between the food simulant (20 mL) and the extraction solvent (5 mL) volumes, the extraction yield was determined. The obtained absolute recovery values (extraction yield) were different depending on the target analyte, ranging from 8 to 110% (Table S2). In this sense, the most polar analytes (such as 4-methylphenol, butyl lactate, and phenol, among others) showed the lowest extraction efficiencies. Moreover, the composition of the studied food simulants (A, B, C, and D1) also affected the observed extraction yield, thus showing that procedural standard calibration (i.e., performing the extraction process in the preparation of the calibration curve) would be necessary in order to compensate for the variation of the extraction yields to obtain accurate results.

### 2.5. Study of Stability of the Target Analytes

In cases where it was necessary to perform the proposed analytical method, including migration tests, VA-LLE, and GC-HRMS analysis, in different working sessions, the stability of the target analytes in the food simulants and in the n-hexane extracts was studied.

In triplicate, a standard solution containing the target analytes at 160 µg L^−1^ in the food simulant was analyzed following the proposed method immediately after its preparation, and after 1, 3, and 5 days of fridge storage (4 °C). The variation in the analytical signal was evaluated, and the obtained results shown that the target analytes were stable during the first 24 h of fridge storage (RSD, <15%). After 1 day of fridge storage, a decrease of the analytical signal was observed for some of the target analytes, such as 3-(trimethoxysilyl)propyl methacrylate, lauryl acrylate, triethoxyvinyl silane, triethyl phosphite, and vinyltrimethoxysilane. Similar results were obtained for all the tested food simulants (A, B, C, and D1).

Moreover, the stability of the target analytes was also tested in the n-hexane extract obtained after the VA-LLE analysis. In triplicate, a standard solution containing the target analytes at 160 µg L^−1^ in the food simulant was subjected to VA-LLE, and the obtained n-hexane extract was analyzed immediately, and after 1, 3, and 5 days of freezer storage (−24 °C). The variation in the analytical signal was evaluated, and the obtained results showed that the target analytes were stable for at least 3 days of freezer storage after the VA-LLE analysis (RSD, <20%). Similar results were obtained for all the tested food simulants (A, B, C, and D1).

### 2.6. Method Validation

In order to validate the proposed methodology, the analytical figures of merit (i.e., linearity, limits of quantification (LOQ), precision, and relative recovery) were evaluated in the studied food simulants (A, B, C, and D1) following the guidelines of the ‘Analytical quality control and method validation procedures for pesticide residues analysis in food and feed‘ (Document No. SANTE/12682/2019) [38]. The obtained analytical figures of merit using food simulant A are shown in Table 2, while those obtained for food simulants B, C, and D1 are shown in Tables S3–S5, respectively.

Linearity comprised at least from 40 to 400 µg L^−1^, obtaining coefficients of determination (R^2^) ≥ 0.98 in all the cases.

The LOQ, defined for practical reasons as the lowest concentration of the calibration curve, was fixed at 40 µg L^−1^ in the food simulant. Although many of the target analytes could be determined in concentrations below the reported LOQ, lower concentration limits were not pursued because the achieved method LOQ enables the evaluation of compliance with their SML [1]. The method LOQ, expressed in mg kg^−1^ of food, depends on the contact surface of the material exposed to the food simulant, considering the conventional ratio of 6 dm^2^ of contact surface per kg of food (see Section 3.4.5). As an example, a migration test performed with 1 dm^2^ of the plastic FCM exposed to 40 mL of food simulant would provide a method LOQ of 0.01 mg kg^−1^, applying the proposed methodology. According to Regulation 10/2011 [1], the SML for IAS range from 0.05 to 60 mg kg^−1^ of food, except for those whose SML states ND (not detected) and for NIAS, which should not be found in concentrations higher than 0.01 mg kg^−1^ [1].

Precision was evaluated in terms of the relative standard deviation (RSD, %) obtained in the analysis of five replicates of standard solutions containing the target analytes at three concentration levels (40, 160, and 400 µg L^−1^). The obtained results ranged from 0.6 to 12.6%, thus showing that satisfactory precision was achieved.

#### 2.6.1. Study of Matrix Effects

In order to validate the method accuracy, the matrix effects were studied by comparing the analytical response obtained in the analysis of solvent standards and matrix-matched standards (spiked samples). In this sense, the relative recovery values (%) obtained in the analysis of spiked and non-spiked field samples, applying the proposed methodology with the four food simulants (A, B, C, and D1), were calculated.

In triplicate, the sample extracts obtained in the migration tests of the field samples (see Section 3.4.1) were spiked with the target analytes at three concentration levels (40, 160, and 400 µg L^−1^), and they were quantified using the proposed procedural standard calibration. The relative recovery values (%) were evaluated by comparing the determined concentration of the target analytes in the spiked and non-spiked samples with the known spiked concentration (solvent standard). The obtained relative recovery values ranged from 81 to 120%, thus showing that there were no matrix effects and, therefore, the proposed procedural standard calibration provided accurate results.

### 2.7. Analysis of Field Samples

The proposed method was applied in the analysis of four field samples made of different plastic FCM (see Section 3.2). The migration tests were performed according to Regulation 10/2011 [1] (see Section 3.4.1).

In the analysis of the field samples, no target analytes were found in concentrations above the method LOQ, thus showing that the tested samples were in accordance with the abovementioned regulation with regard to the studied substances [1]. Moreover, the study of matrix effects discussed above (see Section 2.6.1) demonstrated that there were no matrix effects that could affect the accuracy of the obtained results.

However, it could be interesting to apply the proposed methodology to a larger number of field samples of plastic FCM to obtain data regarding the appearance rate of the studied substances as market surveillance.

## 3. Materials and Methods

### 3.1. Reagents

Analytical standards of the target analytes were purchased from Merck KGaA (Darmstadt, Germany). The migrant substances studied in this paper, and their SML, are summarized in Table 1.

Analytical standards of phenol-^13^C_6_ (CAS 89059-34-7), and bis(2-ethylhexyl) phthalate-D_4_ (CAS 93951-87-2), both from LGC Standards (Bury, UK), were used as internal standards.

Acetone, residue-analysis grade; n-hexane, residue-analysis grade; and methanol, MS-grade, all from VWR International (Radnor, PA, USA), were used as solvents.

Glacial acetic acid, reagent-grade, from Panreac Química (Barcelona, Spain); absolute ethanol, gradient-grade; and water, MS-grade, both from VWR International (Radnor, PA, USA), were used to prepare the food simulants.

### 3.2. Samples

An epoxy-coated tin food can (Sample 1), a drinking bottle made of Tritan copolyester (Sample 2), a disposable glass made of polycarbonate (Sample 3), and a baby feeding bottle made of polypropylene (Sample 4) were analyzed. The samples were obtained directly from the manufacturer, and they had not previously been in contact with food.

### 3.3. Instruments

A Trace 1310 GC system equipped with a TraceGOLD TG-5MS column (30 m, 0.25 mm, 0.25 µm), coupled to a Q-Exactive GC Orbitrap HRMS detector, all from Thermo Fisher Scientific (Waltham, MA, USA), were used for GC-HRMS analysis.

A Conterm laboratory oven, from J.P. Selecta (Barcelona, Spain); an Advanced IR vortex mixer, from VELP Scientifica (Usmate Velate, Italy); and an Allegra X-15R centrifuge, from Beckman Coulter (Indianapolis, IN, USA), were also used.

### 3.4. Proposed Method

#### 3.4.1. Preparation of Samples. Migration Tests

According to Regulation 10/2011 [1], migration tests have to be performed by exposing the plastic FCM in the appropriate food simulant under standardized test conditions, according to its worst foreseeable use.

The food simulants that were considered and can be applied in the proposed method are as follows: food simulant A (ethanol, 10% *v*/*v*), food simulant B (acetic acid, 3% *w*/*v*), food simulant C (ethanol, 20% *v*/*v*), and food simulant D1 (ethanol, 50% *v*/*v*).

The intended use of the analyzed samples and the selected conditions for the migration tests are summarized in Table 3. Briefly, Sample 1, intended to contain preserved vegetables or animal products in an oily medium at room temperature for a long period of time (above 30 days), was tested using food simulant A for 10 days at 60 °C; Sample 2, intended to contain clear drinks, such as water, fruit juice, or energy drinks, was tested using food simulant B for 24 h at 40 °C; Sample 3, intended to contain alcoholic or non-alcoholic beverages, such as wine or beer, was tested using food simulant C for 2 h at 40 °C; and Sample 4, intended to contain milk or milk-based drinks, including reconstituted milk-based on infant formula, was tested using food simulant D1 for 2 hours at 70 °C, followed by 24 h at 40 °C.

Three individual units of each sample were filled with the selected food simulant, sealed with aluminum foil to prevent solvent evaporation, and placed into a laboratory oven for its incubation. After the migration tests, the food simulants were collected and submitted to the subsequent VA-LLE procedure.

It should be noted that the specific conditions for the migration test (food simulant composition, contact time, and contact temperature) depend on the worst foreseeable use of the plastic FCM. The methodology proposed in this paper can be applied to the analysis of any plastic FCM that uses any of the described food simulants (A, B, C, and D1) for its migration test.

#### 3.4.2. Preparation of Standards

Stock individual standard solutions of the target analytes and the internal standards were prepared at 1600 mg L^−1^ in methanol. From these solutions, stock multicomponent solutions containing the target analytes and the internal standards, respectively, at 16 mg L^−1^ were prepared in acetone. These stock standard solutions were stored in a freezer (−24 °C) when necessary until use.

The standard solutions used to prepare the calibration curve, containing the target analytes from 40 to 400 µg L^−1^, were prepared using the four food simulants. Within the working range, a 6-point calibration curve was constructed using the following concentration levels: 40, 80, 160, 240, 320, and 400 µg L^−1^. For this, aliquots of the stock multicomponent standard solution, ranging from 50 to 500 µL, were placed into a series of 20 mL volumetric flasks, and they were filled to the line using food simulant as a solvent. These standard solutions were freshly prepared in each analytical sequence.

#### 3.4.3. Vortex-Assisted Liquid–Liquid Extraction (VA-LLE)

After the migration test, 20 mL of the food simulant were collected and placed into a 50 mL glass centrifuge tube, and 5 mL of n-hexane were added. The mixture was stirred vigorously for 30 s in a vortex mixer (ca. 2000 rpm) in order to enhance the extraction effectiveness, and it was then centrifuged at 3000 rpm for 5 min for phase separation. Finally, 1 mL of the n-hexane extract (supernatant phase) was collected, placed into an injection vial, and spiked with 20 µL of the internal standards solution for subsequent GC-HRMS analysis. Additionally, sample blanks consisting of blank food simulant subjected to VA-LLE were also prepared in triplicate following the same procedure.

The VA-LLE was also applied to the calibration curve (see Section 3.4.2) following the described extraction procedure under the same conditions prior to GC-HRMS analysis (procedural standard calibration).

#### 3.4.4. GC-HRMS Analysis

One microliter of the standard or sample extract was injected into the GC-HRMS system (splitless injection). The inlet temperature was set at 280 °C. The GC operated in constant flow mode at 1.2 mL min^−1^ with helium as a carrier gas, using the following oven temperature programs: 40 °C, held for 5 min; 5 °C min^−1^ up to 315 °C, held for 10 min. The MS transfer line was set at 300 °C. The EI ion source operated at 70 eV, and its temperature was 250 °C. The acquisition was performed in selected ion monitoring (SIM) mode with a resolving power of 30,000 FWHM and a mass accuracy ≤ 5 ppm. The selected ions for signal acquisition and the retention times of the target analytes are shown in Table S1.

#### 3.4.5. Quantification and Confirmation

The quantification of the target analytes was performed by procedural standard calibration with internal standards in the working range, from 40 to 400 µg L^−1^ (6-point calibration curve). The calibration curves were obtained by simple linear regression, with the analytical signal (i.e., ratio between the target analyte peak area and the internal standard peak area) being the dependent variable and the analyte concentration (µg L^−1^) being the independent variable. The internal standard bis(2-ethylhexyl) phthalate-D_4_ was used to quantify benzyl butyl phthalate, bis(2-ethylhexyl) adipate, bis(2-ethylhexyl) phthalate, diallyl phthalate, dibutyl adipate, dibutyl maleate, dibutyl phthalate, diethyl phthalate, diisobutyl phthalate, dimethyl isophthalate, dimethyl terephthalate, and diphenyl phthalate, and the internal standard phenol-^13^C_6_ was used to quantify the other target analytes.

The results, expressed in µg L^−1^ of food simulant, were obtained by interpolation in the calibration curves. In order to evaluate compliance with the SML of Regulation 10/2011 [1], the results needed to be converted to mg kg^−1^ of food. For this, the conventional ratio of 6 dm^2^ of food contact surface per kg of food was considered using the following expression: M = C × (6 V/S) × 10^−3^; ‘M’ being the migration of the target analyte in mg kg^−1^ of food; ‘C’ the obtained concentration of the target analyte in µg L^−1^ of food simulant; ‘V’ the volume of food simulant in L or dm^3^; and ‘S’ the contact surface of the FCM in dm^2^.

The identification and confirmation of the target analytes was performed by comparing the chromatographic peaks obtained for the standards and the samples, considering the following criteria: retention time should not differ by more than 0.1 min, the two selected ions should be detected (quantification ion and confirmation ion) (see Table S1) with mass accuracy ≤2 ppm, and the analyte peaks in the extracted ion chromatogram of the two selected ions must fully overlap.

## 4. Conclusions

In this work, a useful analytical method for the determination of 60 migrant substances, including aldehydes, ketones, phthalates and other plasticizers, phenol derivatives, acrylates, and methacrylates, from plastic FCM using food simulants has been developed and validated. The proposed methodology included migration tests of plastic FCM using food simulants A, B, C, and D1, followed by VA-LLE using only n-hexane as an extraction solvent and GC-Q-Orbitrap HRMS analysis. Migration tests were performed following standardized conditions, according to current Regulation 10/2011, and the experimental variables involved in the vortex-assisted liquid–liquid extraction were studied. The proposed method was then validated using procedural standard calibration, showing satisfactory linearity, method limits of quantification, precision, and relative recovery; it was applied in the analysis of four field samples made of different plastic FCM, showing that the target analytes were in compliance with their SML according to Regulation 10/2011. In this sense, the analytical method addressed in this paper constitutes a fast, useful, and reliable procedure for the analysis of plastic FCM using food simulants for the control of compliance with current European regulations. It should be noted that, in the current market, there is a wide variety of FCM made of different plastics and polymer materials and not all the studied substances, both IAS and NIAS, may be relevant for all the plastic FCM. In this sense, a multi-analyte method such as the one proposed in the present manuscript, could be useful for food control authorities in order to evaluate compliance of plastic FCM with current European regulations as a part of a comprehensive analytical control plan.

## Figures and Tables

**Table 1:** List of target analytes and their specific migration limits (SML).

Analyte	CAS No.	FCM No. ^a^	SML (mg kg^−1^) ^b^
1,4-Butanediol dimethacrylate	2082-81-7	434	0.05
1,4-Dichlorobenzene	106-46-7	217	12
2,2,4-Trimethyl-1,3-pentanediol diisobutyrate	6846-50-0	497	5
2-Ethyl-1-hexanol	104-76-7	209	30
2-Ethylhexyl acrylate	103-11-7	206	0.05
3-(4-Isopropylphenyl)-2-methylpropionaldehyde	103-95-7	-	-
3-(Trimethoxysilyl)propyl methacrylate	2530-85-0	788	0.05
4,4′-Difluorobenzophenone	345-92-6	337	0.05
4-Methylphenol	106-44-5	216	-
4-tert-Butylphenol	98-54-4	186	0.05
Allyl methacrylate	96-05-9	175	0.05
α-Methylstyrene	98-83-9	187	0.05
α-Pinene	80-56-8	155	-
Benzaldehyde	100-52-7	195	-
Benzophenone	119-61-9	286	0.6
Benzyl butyl phthalate	85-68-7	159	30^(1)^
Benzyl methacrylate	2495-37-6	447	6^(2)^
β-Pinene	127-91-3	314	-
Bis(2-ethylhexyl) adipate	103-23-1	207	18^(1)^
Bis(2-ethylhexyl) phthalate	117-81-7	283	1.5^(1)^
Bis(4-chlorophenyl) sulphone	80-07-9	152	0.05
Butyl acrylate	141-32-2	325	6^(3)^
Butyl benzoate	136-60-7	320	-
Butyl lactate	138-22-7	322	-
Butyl methacrylate	97-88-1	184	6^(2)^
Butylated hydroxytoluene	128-37-0	315	3
Camphor	76-22-2	136	-
Cyclohexyl methacrylate	101-43-9	197	0.05
Diallyl phthalate	131-17-9	316	ND
Dibutyl adipate	105-99-7	-	-
Dibutyl maleate	105-76-0	-	-
Dibutyl phthalate	84-74-2	157	0.3^(1)^
Diethyl phthalate	84-66-2	-	-
Diisobutyl phthalate	84-69-5	-	-
Dimethyl isophthalate	1459-93-4	420	0.05
Dimethyl terephthalate	120-61-6	288	-
Diphenyl phthalate	84-62-8	-	-
Diphenyl sulfone	127-63-9	313	3
Divinyl benzene	1321-74-0	405	ND
Ethyl benzoate	93-89-0	172	-
Ethylene glycol dimethacrylate	97-90-5	185	0.05
Etocrylene	5232-99-5	487	0.05
Isobutyl acrylate	106-63-8	218	6^(3)^
Lauryl acrylate	2156-97-0	437	0.05
Methyl benzoate	93-58-3	171	-
Methyl dihydrojasmonate	24851-98-7	-	-
Methyl salicilate	119-36-8	284	30
Octocrylene	6197-30-4	492	0.05
Phenol	108-95-2	241	3
Phenyl methacrylate	2177-70-0	439	6^(2)^
Propyl benzoate	2315-68-6	441	-
Styrene	100-42-5	193	-
tert-Butyl methacrylate	585-07-9	355	6^(2)^
Triethoxyvinylsilane	78-08-0	142	0.05
Triethyl citrate	77-93-0	140	60^(1)^
Triethyl phosphite	122-52-1	293	ND
Trimethylolpropane trimethacrylate	3290-92-4	463	0.05
Tri-n-butyl acetyl citrate	77-90-7	138	60^(1)^
Vinyl laurate	2146-71-6	436	-
Vinyltrimethoxysilane	2768-02-7	453	0.05

^a^ Food contact material (FCM) number according to Regulation 10/2011 [1]. Substances with no FCM No. are not authorized and considered as non-intentionally added substances (NIAS). ^b^ Specific migration limit (SML) according to Regulation 10/2011 [1]. For authorized substances (IAS) with no SML, the applicable limit is 60 mg kg^−1^; and for substances whose SML is ND (not detected) and NIAS, the applicable limit is 0.01 mg kg^−1^. ^(1)^ Group restriction (32): 60 mg kg^−1^ expressed as the sum of the substances. ^(2)^ Group restriction (23): 6 mg kg^−1^ expressed as methacrylic acid. ^(3)^ Group restriction (22): 6 mg kg^−1^ expressed as acrylic acid.

**Table 2:** Analytical figures of merit of the proposed method using food simulant A.

Analyte	Coefficient of Determination (R^2^) ^a^	Relative Recovery (%) ^b^			Precision (RSD, %) ^c^		
		40 µg L^−1^	160 µg L^−1^	400 µg L^−1^	40 µg L^−1^	160 µg L^−1^	400 µg L^−1^
1,4-Butanediol dimethacrylate	0.998	95 ± 6	106 ± 3	101 ± 1	5.8	2.9	1.3
1,4-Dichlorobenzene	0.998	105 ± 9	101 ± 6	99 ± 3	8.7	5.7	3.5
2,2,4-Trimethyl-1,3-pentanediol diisobutyrate	0.999	106 ± 3	97 ± 2	95 ± 2	3.2	2.0	1.7
2-Ethyl-1-hexanol	0.997	92 ± 8	101 ± 4	100 ± 4	8.3	4.1	4.1
2-Ethylhexyl acrylate	0.999	101 ± 8	113 ± 4	99 ± 2	7.5	3.8	1.6
3-(4-Isopropylphenyl)-2-methylpropionaldehyde	0.994	104 ± 3	96 ± 3	101 ± 2	2.5	3.5	2.1
3-(Trimethoxysilyl)propyl methacrylate	0.993	108 ± 5	81 ± 3	83 ± 1	4.8	4.2	1.2
4,4′-Difluorobenzophenone	0.997	89 ± 5	106 ± 3	97 ± 2	6.0	3.3	2.1
4-Methylphenol	0.997	87 ± 5	101 ± 7	103 ± 5	6.0	6.8	5.1
4-tert-Butylphenol	0.997	94 ± 5	103 ± 7	105 ± 5	5.0	6.4	4.7
Allyl methacrylate	0.996	105 ± 10	99 ± 2	101 ± 4	9.5	2.4	4.1
α-Methylstyrene	0.994	103 ± 9	102 ± 3	108 ± 4	8.6	3.0	3.8
α-Pinene	0.993	100 ± 4	97 ± 2	100 ± 3	4.4	2.5	2.7
Benzaldehyde	0.996	90 ± 7	99 ± 4	102 ± 4	8.3	3.7	3.6
Benzophenone	0.997	92 ± 7	106 ± 4	102 ± 7	8.0	4.1	7.1
Benzyl butyl phthalate	0.994	97 ± 4	104 ± 5	102 ± 1	4.1	5.3	0.9
Benzyl methacrylate	0.999	94 ± 7	106 ± 5	102 ± 4	7.6	5.2	4.4
β-Pinene	0.996	104 ± 6	101 ± 3	100 ± 4	6.0	2.7	3.6
Bis(2-ethylhexyl) adipate	0.988	82 ± 4	103 ± 8	111 ± 11	4.6	7.8	10.0
Bis(2-ethylhexyl) phthalate	0.992	99 ± 4	103 ± 5	113 ± 8	4.4	4.8	7.2
Bis(4-chlorophenyl) sulphone	0.997	90 ± 7	95 ± 8	95 ± 2	8.1	8.6	2.1
Butyl acrylate	0.998	106 ± 9	94 ± 2	104 ± 2	8.7	2.3	2.2
Butyl benzoate	0.999	92 ± 8	107 ± 5	100 ± 1	8.3	4.4	0.8
Butyl lactate	0.996	96 ± 3	101 ± 1	100 ± 6	3.6	0.7	6.0
Butyl methacrylate	0.991	98 ± 10	99 ± 3	103 ± 4	10.0	3.2	4.1
Butylated hydroxytoluene	0.997	94 ± 5	106 ± 5	100 ± 1	5.1	4.7	1.3
Camphor	0.997	93 ± 7	112 ± 3	106 ± 2	7.6	2.8	2.2
Cyclohexyl methacrylate	0.997	100 ± 9	113 ± 7	103 ± 1	9.3	6.2	1.3
Diallyl phthalate	0.998	97 ± 7	98 ± 3	100 ± 1	6.8	3.5	0.6
Dibutyl adipate	0.998	90 ± 5	100 ± 5	100 ± 6	6.0	5.1	6.0
Dibutyl maleate	0.993	100 ± 5	92 ± 5	101 ± 1	5.1	5.7	1.0
Dibutyl phthalate	0.997	99 ± 6	105 ± 5	100 ± 1	6.1	4.7	0.8
Diethyl phthalate	0.999	98 ± 2	104 ± 4	96 ± 2	2.5	3.7	1.9
Diisobutyl phthalate	0.997	92 ± 7	105 ± 5	96 ± 1	7.6	4.8	0.9
Dimethyl isophthalate	0.994	92 ± 9	101 ± 2	99 ± 1	9.4	1.8	1.3
Dimethyl terephthalate	0.994	88 ± 6	99 ± 4	98 ± 1	7.0	3.7	1.1
Diphenyl phthalate	0.998	91 ± 4	96 ± 6	102 ± 1	4.6	6.5	0.8
Diphenyl sulphone	0.994	88 ± 9	105 ± 4	97 ± 8	10.2	3.8	8.1
Divinyl benzene	0.997	104 ± 7	107 ± 5	108 ± 3	6.8	4.9	2.5
Ethyl benzoate	0.995	96 ± 8	106 ± 1	106 ± 7	8.4	1.4	6.4
Ethylene glycol dimethacrylate	0.999	96 ± 6	103 ± 5	101 ± 1	6.6	4.4	1.4
Etocrylene	0.998	93 ± 6	97 ± 6	104 ± 9	6.5	6.0	8.5
Isobutyl acrylate	0.997	93 ± 4	99 ± 3	98 ± 2	4.5	3.2	2.1
Lauryl acrylate	0.989	94 ± 8	113 ± 7	99 ± 3	8.6	6.2	3.4
Methyl benzoate	0.990	93 ± 8	103 ± 3	102 ± 6	8.5	3.3	6.0
Methyl dihydrojasmonate	0.993	99 ± 6	97 ± 5	102 ± 1	6.1	5.6	1.0
Methyl salicylate	0.996	95 ± 6	102 ± 4	107 ± 6	6.3	3.5	5.9
Octocrylene	0.994	102 ± 3	91 ± 7	109 ± 5	2.6	7.6	4.8
Phenol	0.990	93 ± 5	97 ± 5	106 ± 4	5.1	4.8	4.0
Phenyl methacrylate	0.999	96 ± 7	103 ± 2	105 ± 3	7.0	1.9	3.1
Propyl benzoate	0.998	99 ± 8	108 ± 2	101 ± 1	8.3	2.0	1.3
Styrene	0.995	100 ± 3	96 ± 3	100 ± 2	2.6	3.0	2.1
tert-Butyl methacrylate	0.992	103 ± 5	100 ± 2	96 ± 2	4.4	2.5	1.8
Triethoxyvinylsilane	0.995	98 ± 8	96 ± 6	85 ± 3	8.7	6.5	3.8
Triethyl citrate	0.999	99 ± 4	91 ± 5	98 ± 9	4.4	5.7	8.9
Triethyl phosphite	0.992	81 ± 1	99 ± 4	82 ± 4	1.1	3.6	4.6
Trimethylolpropane trimethacrylate	0.994	106 ± 4	93 ± 4	104 ± 1	3.4	4.9	0.9
Tri-n-butyl acetyl citrate	0.995	101 ± 4	88 ± 7	103 ± 10	4.2	7.5	9.3
Vinyl laurate	0.999	90 ± 6	110 ± 5	100 ± 2	6.9	4.4	1.8
Vinyltrimethoxysilane	0.999	101 ± 11	100 ± 9	98 ± 10	11.1	8.8	10.6

^a^ Coefficient of determination (R^2^) of the calibration curve in the range 40–400 µg L^−1^, n = 6. ^b^ Relative recovery values (%) obtained in the analysis of Sample 1, expressed as the mean value of three replicates ± standard deviation. ^c^ Precision expressed as relative standard deviation (RSD, %), n = 5.

**Table 3:** Migration test conditions for the analyzed samples according to Regulation 10/2011 [1].

Sample	Food Category of Intended Use	Worst Foreseeable Food Contact Time (t) and Temperature (T)	Selected Conditions for Migration Test
1	Preserved vegetables or preserved animal products (fish, meat) in an oily medium	Above 30 days, 20 °C < T ≤ 40 °C	Food simulant A, 10 days at 60 °C
2	Clear drinks: Water, ciders, fruit or vegetable juices, infusions, coffee, tea, energy drinks, etc.	6 hours < t ≤ 24 h, 20 °C < T ≤ 40 °C	Food simulant B, 24 h at 40 °C
3	Alcoholic beverages of an alcoholic strength <20% (wine, beer, etc.) or clear drinks	1 hour < t ≤ 2 h, 20 °C < T ≤ 40 °C	Food simulant C, 2 h at 40 °C
4	Milk and milk-based drinks whole, partly dried and skimmed, or partly skimmed	6 hours < t ≤ 24 h, 20 °C < T ≤ 40 °C, including a hot filling up to 70 °C	Food simulant D1, 2 hours at 70 °C and 24 h at 40 °C

## Data Availability

Data are available from corresponding author upon reasonable request.

## Supplementary Materials

The following are available online. Table S1: Selected ions (*m*/*z*) for HRMS acquisition and retention time (RT) of the target analytes, Table S2: Extraction efficiencies of the target analytes from the studied food simulants (A, B, C, and D1), Table S3: Analytical figures of merit of the proposed method using food simulant B, Table S4: Analytical figures of merit of the proposed method using food simulant C, Table S5: Analytical figures of merit of the proposed method using food simulant D1.
