# Supplementary material for: Determination of 60 Migrant Substances in Plastic Food Contact Materials by Vortex-Assisted Liquid-Liquid Extraction and GC-Q-Orbitrap HRMS
Source: Molecules. 2021 Dec 16;26(24):7640. doi: 10.3390/molecules26247640 (PMC8703817; doi:10.3390/molecules26247640)
Supplement: Supplementary file 1 [file molecules-26-07640-s001.zip › molecules-1516301-supplementary.pdf]

SUPPLEMENTARY MATERIAL

Determination of 60 migrant substances in plastic food contact materials by vortex-assisted liquid-liquid extraction and GC-Q-Orbitrap HRMS

Pablo Miralles [1,2], Vicent Yusà [1,3], Yovana Sanchís [1,3], Clara Coscollà [1,*]

[1] Foundation for the Promotion of Health and Biomedical Research in the Valencian Region, FISABIO-Public Health, Av. Catalunya 21, 46020 Valencia, Spain

[2] Department of Analytical Chemistry, University of Valencia, Av. Doctor Moliner 50, 46100 Burjassot, Spain

[3] Public Health Laboratory of Valencia, Av. Catalunya 21, 46020 Valencia, Spain

[*] Corresponding author.

E-mail address: coscolla_cla@gva.es (C. Coscollà)

Tables	Page
**Table S1**. Selected ions (m/z) for HRMS acquisition and retention time (RT) of the target analytes.	2
**Table S2**. Extraction efficiencies of the target analytes from the studied food simulants (A, B, C, and D1).	4
**Table S3**. Analytical figures of merit of the proposed method using food simulant B.	7
**Table S4**. Analytical figures of merit of the proposed method using food simulant C.	10
**Table S5**. Analytical figures of merit of the proposed method using food simulant D1.	13

Table S1. Selected ions (m/z) for HRMS acquisition and retention time (RT) of the target analytes.

Analyte	Quantification ion (m/z)	Confirmation ion (m/z)	RT (min)
1,4-Butanediol dimethacrylate	69.03349	126.06753	28.24
1,4-Dichlorobenzene	145.96846	147.96551	13.29
2,2,4-Trimethyl-1,3-pentanediol diisobutyrate	71.04914	43.05423	29.45
2-Ethyl-1-hexanol	57.06988	83.08553	13.91
2-Ethylhexyl acrylate	55.05423	70.07770	20.10
3-(4-Isopropylphenyl)-2-methylpropionaldehyde	133.10118	105.06988	26.37
3-(Trimethoxysilyl)propyl methacrylate	121.03157	91.02103	25.53
4,4'-Difluorobenzophenone	123.02407	218.05377	29.50
4-Methylphenol	107.04914	108.05697	15.40
4-tert-Butylphenol	135.08044	107.04914	21.89
Allyl methacrylate	69.03349	111.04409	8.22
α-Methylstyrene	118.07770	117.06988	12.18
α-Pinene	93.06988	91.05423	10.44
Benzaldehyde	105.03349	77.03858	11.45
Benzophenone	105.03349	182.07262	30.37
Benzyl butyl phthalate	149.02332	91.05423	44.12
Benzyl methacrylate	131.08548	91.05423	23.01
β-Pinene	93.06988	91.05423	12.02
Bis(2-ethylhexyl) adipate	129.05462	111.04406	44.96
Bis(2-ethylhexyl) phthalate	149.02332	167.03389	47.17
Bis(2-ethylhexyl) phthalate-D_4*	153.04840	171.05895	47.13
Bis(4-chlorophenyl) sulphone	158.96659	285.96188	42.37
Butyl acrylate	55.01784	73.02841	9.17
Butyl benzoate	105.03349	123.04406	24.14
Butyl lactate	45.03349	85.06479	13.11
Butyl methacrylate	87.04406	41.03858	12.18
Butylated hydroxytoluene	205.15865	220.18214	27.44
Camphor	95.08553	108.09335	17.67
Cyclohexyl methacrylate	87.04412	67.05423	19.42
Diallyl phthalate	149.02332	104.02567	32.82
Dibutyl adipate	129.05462	111.04406	33.37

Dibutyl maleate	99.00774	117.01826	28.15
Dibutyl phthalate	149.02332	150.02667	37.21
Diethyl phthalate	149.02332	176.04680	29.40
Diisobutyl phthalate	149.02332	104.02567	35.32
Dimethyl isophthalate	163.03897	135.04406	27.56
Dimethyl terephthalate	163.03897	135.04405	27.26
Diphenyl phthalate	225.05455	77.03858	47.17
Diphenyl sulfone	125.00561	97.01071	36.48
Divinyl benzene	130.07770	115.05423	16.63
Ethyl benzoate	105.03349	77.03858	18.39
Ethylene glycol dimethacrylate	113.05975	69.03349	22.45
Etocrylene	204.08073	232.07567	40.95
Isobutyl acrylate	55.01784	73.02841	7.65
Lauryl acrylate	55.05423	83.08553	31.85
Methyl benzoate	105.03349	77.03858	16.00
Methyl dihydrojasmonate	83.04914	156.07810	30.84
Methyl salicylate	120.02058	92.02567	19.04
Octocrylene	248.07061	232.07569	48.89
Phenol	94.04132	66.04640	12.25
Phenol-$^{13}C_6$*	100.06149	71.06321	12.24
Phenyl methacrylate	134.07262	69.03349	20.07
Propyl benzoate	105.03349	123.04406	21.30
Styrene	104.06205	78.04640	8.87
tert-Butyl methacrylate	87.04406	57.06988	7.65
Tri-n-butyl acetyl citrate	129.01819	185.08070	42.61
Triethoxyvinylsilane	145.06793	135.04718	11.28
Triethyl citrate	157.04955	115.03902	30.92
Triethyl phosphite	82.98927	111.02060	10.25
Trimethylolpropane trimethacrylate	69.03349	253.14349	38.36
Vinyl laurate	183.17429	95.08553	28.90
Vinyltrimethoxysilane	91.02103	121.03157	5.49

* Substances used as internal standards.

Table S2. Extraction efficiencies of the target analytes from the studied food simulants (A, B, C, and D1).

Analyte*	Extraction efficiency (%)[a]			
	Food simulant A	Food simulant B	Food simulant C	Food simulant D1
1,4-Butanediol dimethacrylate	101 ± 3	90 ± 9	100 ± 9	73 ± 4
1,4-Dichlorobenzene	110 ± 2	99 ± 8	90 ± 1	93 ± 3
2,2,4-Trimethyl-1,3-pentanediol diisobutyrate	103 ± 3	105 ± 12	104 ± 5	100 ± 3
2-Ethyl-1-hexanol	90 ± 2	81 ± 8	84 ± 2	26 ± 1
2-Ethylhexyl acrylate	80 ± 2	79 ± 3	83 ± 1	93 ± 2
3-(4-Isopropylphenyl)-2-methylpropionaldehyde	104 ± 5	102 ± 10	100 ± 2	54 ± 1
3-(Trimethoxysilyl)propyl methacrylate	95 ± 4	-	106 ± 5	50 ± 2
4,4′-Difluorobenzophenone	102 ± 5	105 ± 9	99 ± 6	97 ± 3
4-Methylphenol	12 ± 2	14 ± 2	11 ± 1	8 ± 1
4-tert-Butylphenol	107 ± 4	104 ± 10	82 ± 3	16 ± 1
Allyl methacrylate	102 ± 6	98 ± 8	90 ± 2	69 ± 4
α-Methylstyrene	100 ± 4	104 ± 7	96 ± 3	104 ± 5
α-Pinene	88 ± 7	73 ± 5	60 ± 2	73 ± 4
Benzaldehyde	69 ± 5	52 ± 6	47 ± 3	16 ± 1
Benzophenone	103 ± 3	102 ± 8	104 ± 4	59 ± 2
Benzyl butyl phthalate	101 ± 3	105 ± 9	110 ± 4	104 ± 5
Benzyl methacrylate	104 ± 4	108 ± 13	103 ± 3	89 ± 3
β-Pinene	100 ± 7	85 ± 5	73 ± 2	87 ± 2
Bis(2-ethylhexyl) adipate	101 ± 4	94 ± 3	99 ± 5	105 ± 9

Bis(2-ethylhexyl) phthalate	99 ± 7	94 ± 9	95 ± 7	98 ± 11
Bis(4-chlorophenyl) sulphone	101 ± 3	93 ± 7	105 ± 9	57 ± 2
Butyl acrylate	84 ± 3	77 ± 5	74 ± 2	60 ± 1
Butyl benzoate	102 ± 5	106 ± 12	101 ± 3	87 ± 2
Butyl lactate	24 ± 2	25 ± 4	20 ± 3	-
Butyl methacrylate	102 ± 2	107 ± 8	99 ± 5	98 ± 4
Butylated hydroxytoluene	104 ± 6	109 ± 10	102 ± 5	94 ± 2
Camphor	100 ± 3	99 ± 9	97 ± 2	57 ± 3
Cyclohexyl methacrylate	105 ± 3	98 ± 7	101 ± 3	94 ± 2
Diallyl phthalate	100 ± 5	102 ± 9	95 ± 4	49 ± 2
Dibutyl adipate	99 ± 3	103 ± 8	107 ± 7	84 ± 6
Dibutyl maleate	97 ± 8	98 ± 9	100 ± 5	73 ± 4
Dibutyl phthalate	97 ± 4	95 ± 2	99 ± 9	88 ± 4
Diethyl phthalate	99 ± 5	92 ± 7	98 ± 4	63 ± 2
Diisobutyl phthalate	96 ± 4	97 ± 6	99 ± 5	95 ± 3
Dimethyl isophthalate	100 ± 3	86 ± 9	101 ± 4	47 ± 2
Dimethyl terephthalate	103 ± 7	89 ± 8	94 ± 4	48 ± 4
Diphenyl phthalate	104 ± 9	96 ± 9	102 ± 5	75 ± 5
Diphenyl sulphone	99 ± 3	93 ± 3	98 ± 2	31 ± 2
Divinyl benzene	104 ± 4	89 ± 8	101 ± 5	105 ± 5
Ethyl benzoate	100 ± 3	102 ± 9	103 ± 2	69 ± 7
Ethylene glycol dimethacrylate	100 ± 4	92 ± 8	97 ± 3	42 ± 2
Etocrylene	95 ± 3	91 ± 4	98 ± 9	58 ± 3

Isobutyl acrylate	85 ± 3	76 ± 6	72 ± 3	59 ± 4
Lauryl acrylate	97 ± 6	97 ± 9	102 ± 3	99 ± 2
Methyl benzoate	98 ± 4	88 ± 7	85 ± 2	48 ± 2
Methyl dihydrojasmonate	102 ± 5	94 ± 6	96 ± 7	65 ± 2
Methyl salicylate	99 ± 3	101 ± 5	97 ± 5	79 ± 3
Octocrylene	101 ± 5	81 ± 6	97 ± 5	87 ± 3
Phenol	12 ± 2	8 ± 1	-	-
Phenyl methacrylate	95 ± 5	93 ± 4	93 ± 5	89 ± 2
Propyl benzoate	98 ± 3	96 ± 8	98 ± 3	73 ± 3
Styrene	91 ± 7	75 ± 6	85 ± 3	71 ± 2
tert-Butyl methacrylate	102 ± 6	95 ± 9	81 ± 1	79 ± 4
Triethoxyvinylsilane	82 ± 8	-	72 ± 4	77 ± 2
Triethyl citrate	90 ± 7	80 ± 3	77 ± 5	-
Triethyl phosphite	15 ± 1	-	-	48 ± 2
Trimethylolpropane trimethacrylate	103 ± 4	94 ± 3	99 ± 5	96 ± 4
Tri-n-butyl acetyl citrate	100 ± 5	84 ± 6	97 ± 3	-
Vinyl laurate	96 ± 7	85 ± 8	86 ± 6	72 ± 5
Vinyltrimethoxysilane	52 ± 5	-	97 ± 2	67 ± 3

[a] Extraction efficiencies expressed as the mean value of three replicates ± standard deviation.

* Analytes with no data did not meet the validation criteria of Document No. SANTE/12682/2019 [38].

Table S3. Analytical figures of merit of the proposed method in food simulant B.

Analyte*	Coefficient of	Relative recovery (%)[b]	Precision (RSD, %)[c]

	determination (R^2)[a]	40 µg L^{-1}	160 µg L^{-1}	400 µg L^{-1}	40 µg L^{-1}	160 µg L^{-1}	400 µg L^{-1}
1,4-Butanediol dimethacrylate	0.995	102 ± 4	100 ± 8	85 ± 4	4.0	7.5	4.6
1,4-Dichlorobenzene	0.988	98 ± 6	101 ± 9	104 ± 6	6.4	9.2	6.2
2,2,4-Trimethyl-1,3-pentanediol diisobutyrate	0.999	96 ± 7	101 ± 8	81 ± 3	6.9	8.1	3.9
2-Ethyl-1-hexanol	0.999	89 ± 7	96 ± 8	90 ± 5	8.4	8.5	5.2
2-Ethylhexyl acrylate	0.996	89 ± 4	100 ± 9	93 ± 6	5.0	9.1	5.9
3-(4-Isopropylphenyl)-2-methylpropionaldehyde	0.998	101 ± 8	117 ± 11	115 ± 1	7.9	9.2	0.9
3-(Trimethoxysilyl)propyl methacrylate	-	-	-	-	-	-	-
4,4′-Difluorobenzophenone	0.997	105 ± 6	104 ± 6	114 ± 1	6.0	5.5	1.0
4-Methylphenol	0.998	92 ± 4	106 ± 8	99 ± 5	3.9	7.7	4.7
4-tert-Butylphenol	0.996	96 ± 3	100 ± 6	104 ± 6	3.6	6.5	6.0
Allyl methacrylate	0.995	88 ± 9	94 ± 8	85 ± 5	10.5	8.2	6.2
α-Methylstyrene	0.988	99 ± 6	105 ± 6	108 ± 2	6.3	5.9	1.6
α-Pinene	0.988	94 ± 6	99 ± 10	96 ± 6	6.0	9.7	6.6
Benzaldehyde	0.992	91 ± 9	106 ± 7	103 ± 4	10.5	6.9	3.5
Benzophenone	0.991	104 ± 6	102 ± 5	112 ± 1	5.8	4.8	1.2
Benzyl butyl phthalate	0.997	102 ± 4	105 ± 2	115 ± 2	3.8	1.8	1.6
Benzyl methacrylate	0.996	109 ± 7	99 ± 6	112 ± 7	6.8	6.0	6.7
β-Pinene	0.984	96 ± 5	98 ± 9	98 ± 7	4.9	9.6	7.0
Bis(2-ethylhexyl) adipate	0.996	108 ± 4	107 ± 8	110 ± 5	3.3	7.3	4.2
Bis(2-ethylhexyl) phthalate	0.998	101 ± 5	105 ± 6	120 ± 4	5.1	6.0	3.5

Bis(4-chlorophenyl) sulphone	0.997	93 ± 7	112 ± 3	96 ± 9	7.6	2.5	9.1
Butyl acrylate	0.998	96 ± 8	97 ± 10	90 ± 5	8.4	9.9	5.4
Butyl benzoate	0.996	93 ± 7	105 ± 3	107 ± 2	7.4	2.9	2.3
Butyl lactate	0.986	99 ± 2	88 ± 4	103 ± 4	2.4	5.1	4.3
Butyl methacrylate	0.995	88 ± 3	104 ± 6	87 ± 5	3.7	5.5	5.7
Butylated hydroxytoluene	0.996	99 ± 7	99 ± 9	104 ± 5	7.2	8.7	5.1
Camphor	0.998	89 ± 8	105 ± 9	97 ± 6	9.3	8.9	5.9
Cyclohexyl methacrylate	0.994	94 ± 5	103 ± 8	86 ± 5	5.0	7.4	5.3
Diallyl phthalate	0.989	109 ± 7	108 ± 6	111 ± 7	6.0	5.1	5.9
Dibutyl adipate	0.997	108 ± 7	102 ± 4	105 ± 5	6.2	3.5	4.7
Dibutyl maleate	0.988	112 ± 5	107 ± 9	116 ± 3	4.3	8.8	2.5
Dibutyl phthalate	0.993	110 ± 6	104 ± 6	113 ± 7	5.2	5.7	6.1
Diethyl phthalate	0.995	105 ± 5	117 ± 4	114 ± 2	4.7	3.7	2.0
Diisobutyl phthalate	0.996	106 ± 5	108 ± 4	112 ± 9	4.4	3.5	7.7
Dimethyl isophthalate	0.995	101 ± 8	111 ± 6	100 ± 2	7.6	5.8	1.9
Dimethyl terephthalate	0.994	102 ± 7	109 ± 7	102 ± 7	7.2	6.4	7.3
Diphenyl phthalate	0.994	97 ± 5	106 ± 5	105 ± 1	4.9	4.6	1.4
Diphenyl sulphone	0.995	106 ± 3	103 ± 9	111 ± 8	2.4	8.4	7.0
Divinyl benzene	0.986	99 ± 6	107 ± 10	107 ± 6	6.0	9.6	6.0
Ethyl benzoate	0.998	90 ± 8	108 ± 12	101 ± 5	9.1	11.0	4.8
Ethylene glycol dimethacrylate	0.989	94 ± 4	113 ± 12	113 ± 2	4.2	10.3	1.4
Etocrylene	0.991	110 ± 3	110 ± 6	105 ± 7	3.0	5.7	6.8
Isobutyl acrylate	0.987	91 ± 8	102 ± 4	86 ± 5	8.3	3.8	5.8

Lauryl acrylate	0.993	106 ± 6	100 ± 11	96 ± 4	5.5	11.4	3.7
Methyl benzoate	0.999	87 ± 3	108 ± 10	100 ± 6	3.4	9.2	6.3
Methyl dihydrojasmonate	0.989	109 ± 4	98 ± 11	109 ± 5	3.7	11.0	4.9
Methyl salicylate	0.997	96 ± 5	103 ± 7	102 ± 1	5.5	6.7	1.0
Octocrylene	0.993	111 ± 3	113 ± 4	103 ± 3	2.5	3.9	3.4
Phenol	0.992	101 ± 5	103 ± 4	101 ± 8	4.9	4.3	7.5
Phenyl methacrylate	0.998	103 ± 5	101 ± 7	115 ± 7	4.5	6.6	5.9
Propyl benzoate	0.998	98 ± 8	105 ± 11	102 ± 2	8.4	10.1	1.7
Styrene	0.997	92 ± 6	105 ± 5	103 ± 6	6.6	5.2	6.2
tert-Butyl methacrylate	0.996	90 ± 7	103 ± 9	101 ± 6	7.9	8.7	5.9
Triethoxyvinylsilane	-	-	-	-	-	-	-
Triethyl citrate	0.992	100 ± 4	92 ± 5	106 ± 1	4.5	5.4	0.9
Triethyl phosphite	-	-	-	-	-	-	-
Trimethylolpropane trimethacrylate	0.991	104 ± 6	91 ± 4	85 ± 3	5.6	4.4	3.7
Tri-n-butyl acetyl citrate	0.995	105 ± 3	106 ± 5	104 ± 2	3.1	4.7	1.6
Vinyl laurate	0.990	108 ± 6	103 ± 4	108 ± 8	5.3	3.5	7.1
Vinyltrimethoxysilane	-	-	-	-	-	-	-

[a] Coefficient of determination (R^2) of the calibration curve in the range 40-400 μg L^{-1}, n=6.

[b] Relative recovery values (%) obtained in the analysis of Sample 2, expressed as the mean value of three replicates ± standard deviation.

[c] Precision expressed as relative standard deviation (RSD,%), n=5.

* Analytes with no data did not meet the validation criteria of Document No. SANTE/12682/2019 [38].

Table S4. Analytical figures of merit of the proposed method in food simulant C.

Analyte*	Coefficient of determination (R^2)[a]	Relative recovery (%)[b]			Precision (RSD, %)[c]		
		40 µg L^{-1}	160 µg L^{-1}	400 µg L^{-1}	40 µg L^{-1}	160 µg L^{-1}	400 µg L^{-1}
1,4-Butanediol dimethacrylate	0.990	102 ± 1	106 ± 11	101 ± 7	1.0	10.7	7.2
1,4-Dichlorobenzene	0.993	106 ± 7	117 ± 5	113 ± 12	7.0	4.4	10.4
2,2,4-Trimethyl-1,3-pentanediol diisobutyrate	0.994	99 ± 1	109 ± 13	102 ± 8	1.2	12.0	8.4
2-Ethyl-1-hexanol	0.997	110 ± 8	110 ± 7	103 ± 5	7.0	6.3	5.0
2-Ethylhexyl acrylate	0.997	93 ± 4	93 ± 4	103 ± 9	4.1	4.8	8.8
3-(4-Isopropylphenyl)-2-methylpropionaldehyde	0.989	99 ± 4	105 ± 8	99 ± 6	4.1	7.9	5.7
3-(Trimethoxysilyl)propyl methacrylate	0.989	100 ± 3	98 ± 7	107 ± 4	3.1	7.1	3.9
4,4′-Difluorobenzophenone	0.991	104 ± 2	109 ± 12	104 ± 6	1.6	11.1	6.2
4-Methylphenol	0.998	102 ± 7	95 ± 3	98 ± 7	6.5	2.9	6.7
4-tert-Butylphenol	0.994	106 ± 3	98 ± 7	99 ± 9	3.3	6.9	9.0
Allyl methacrylate	0.997	113 ± 3	101 ± 6	106 ± 1	2.4	5.5	1.1
α-Methylstyrene	0.997	109 ± 5	114 ± 7	108 ± 9	4.3	6.0	8.1
α-Pinene	0.995	98 ± 2	106 ± 8	99 ± 8	2.0	7.8	8.4
Benzaldehyde	0.998	112 ± 4	105 ± 5	102 ± 4	3.4	4.9	3.7
Benzophenone	0.992	99 ± 1	108 ± 8	103 ± 7	1.2	7.5	6.8
Benzyl butyl phthalate	0.989	106 ± 2	92 ± 5	99 ± 7	2.2	4.9	6.8
Benzyl methacrylate	0.982	97 ± 3	96 ± 9	103 ± 12	3.4	9.8	11.9
β-Pinene	0.994	101 ± 3	112 ± 7	101 ± 10	3.1	6.6	10.3

Bis(2-ethylhexyl) adipate	0.991	99 ± 1	85 ± 4	89 ± 5	1.5	4.4	5.6
Bis(2-ethylhexyl) phthalate	0.991	101 ± 2	89 ± 4	93 ± 5	2.2	4.2	5.9
Bis(4-chlorophenyl) sulphone	0.989	98 ± 3	91 ± 3	95 ± 3	2.9	2.9	3.0
Butyl acrylate	0.996	93 ± 8	96 ± 2	101 ± 5	8.6	2.1	4.5
Butyl benzoate	0.990	101 ± 5	102 ± 9	101 ± 7	5.1	9.2	6.5
Butyl lactate	0.990	94 ± 5	107 ± 4	105 ± 3	5.3	3.7	2.8
Butyl methacrylate	0.995	113 ± 2	110 ± 7	102 ± 6	1.9	6.3	6.2
Butylated hydroxytoluene	0.983	107 ± 1	107 ± 7	101 ± 9	0.9	6.5	8.5
Camphor	0.987	100 ± 3	106 ± 10	95 ± 11	2.9	9.2	11.2
Cyclohexyl methacrylate	0.992	94 ± 7	94 ± 8	91 ± 7	7.0	8.8	8.1
Diallyl phthalate	0.996	96 ± 2	106 ± 10	102 ± 2	1.9	9.3	2.2
Dibutyl adipate	0.992	100 ± 3	99 ± 10	95 ± 3	2.6	10.0	2.8
Dibutyl maleate	0.990	96 ± 2	105 ± 10	112 ± 5	1.7	9.4	4.4
Dibutyl phthalate	0.984	103 ± 3	98 ± 8	101 ± 3	3.2	8.7	3.1
Diethyl phthalate	0.990	103 ± 2	101 ± 10	103 ± 6	2.2	10.1	5.9
Diisobutyl phthalate	0.993	98 ± 4	97 ± 6	101 ± 4	4.6	6.5	3.5
Dimethyl isophthalate	0.990	99 ± 1	109 ± 6	102 ± 7	1.1	5.2	7.1
Dimethyl terephthalate	0.987	100 ± 2	101 ± 9	106 ± 3	1.6	9.4	3.3
Diphenyl phthalate	0.983	103 ± 2	85 ± 2	91 ± 6	1.5	2.0	6.3
Diphenyl sulphone	0.994	93 ± 6	105 ± 11	101 ± 3	6.1	10.1	2.9
Divinyl benzene	0.999	93 ± 7	101 ± 4	100 ± 6	7.2	3.5	6.5
Ethyl benzoate	0.994	87 ± 2	103 ± 13	100 ± 12	2.3	12.2	11.6
Ethylene glycol dimethacrylate	0.993	93 ± 9	92 ± 10	99 ± 9	9.9	10.5	9.3

Etocrylene	0.989	102 ± 3	91 ± 2	100 ± 5	3.2	2.5	4.5
Isobutyl acrylate	0.994	120 ± 6	101 ± 8	102 ± 7	4.9	7.4	6.7
Lauryl acrylate	0.987	103 ± 7	117 ± 3	100 ± 4	7.0	2.9	4.3
Methyl benzoate	0.997	110 ± 7	100 ± 8	106 ± 8	6.3	7.9	8.0
Methyl dihydrojasmonate	0.990	100 ± 5	94 ± 7	102 ± 4	5.0	6.7	3.7
Methyl salicylate	0.994	89 ± 5	109 ± 9	86 ± 7	5.6	8.6	7.7
Octocrylene	0.987	106 ± 1	82 ± 2	90 ± 5	1.0	2.8	5.3
Phenol	-	-	-	-	-	-	-
Phenyl methacrylate	0.990	94 ± 4	104 ± 13	98 ± 9	4.1	12.6	8.9
Propyl benzoate	0.992	93 ± 5	99 ± 12	96 ± 9	5.9	12.2	9.6
Styrene	0.992	93 ± 1	100 ± 5	98 ± 5	1.5	4.9	5.2
tert-Butyl methacrylate	0.993	107 ± 6	97 ± 9	108 ± 4	5.4	8.8	3.7
Triethoxyvinylsilane	0.995	118 ± 5	100 ± 6	96 ± 7	4.5	5.6	7.2
Triethyl citrate	0.990	104 ± 5	101 ± 5	88 ± 6	5.3	4.8	6.8
Triethyl phosphite	-	-	-	-	-	-	-
Trimethylolpropane trimethacrylate	0.989	106 ± 1	90 ± 6	92 ± 3	1.1	6.2	2.8
Tri-n-butyl acetyl citrate	0.997	113 ± 5	86 ± 6	98 ± 3	4.3	6.7	3.6
Vinyl laurate	0.980	115 ± 1	100 ± 8	107 ± 6	1.1	8.1	5.3
Vinyltrimethoxysilane	0.997	98 ± 5	93 ± 8	99 ± 6	5.1	8.6	6.1

[a] Coefficient of determination (R^2) of the calibration curve in the range 40-400 μg L^{-1}, n=6.

[b] Relative recovery values (%) obtained in the analysis of Sample 3, expressed as the mean value of three replicates ± standard deviation.

[c] Precision expressed as relative standard deviation (RSD,%), n=5.

* Analytes with no data did not meet the validation criteria of Document No. SANTE/12682/2019 [38].

Table S5. Analytical figures of merit of the proposed method in food simulant D1.

Analyte*	Coefficient of determination (R^2)[a]	Relative recovery (%)[b]			Precision (RSD, %)[c]		
		40 μg L^{-1}	160 μg L^{-1}	400 μg L^{-1}	40 μg L^{-1}	160 μg L^{-1}	400 μg L^{-1}
1,4-Butanediol dimethacrylate	0.995	93 ± 2	82 ± 6	95 ± 5	2.1	6.7	5.1
1,4-Dichlorobenzene	0.990	104 ± 7	116 ± 1	108 ± 4	6.3	1.1	3.4
2,2,4-Trimethyl-1,3-pentanediol diisobutyrate	0.996	99 ± 4	103 ± 2	105 ± 3	3.8	2.2	3.2
2-Ethyl-1-hexanol	0.997	103 ± 2	91 ± 1	110 ± 3	1.9	1.3	2.4
2-Ethylhexyl acrylate	0.992	94 ± 4	112 ± 2	101 ± 3	3.9	1.9	3.1
3-(4-Isopropylphenyl)-2-methylpropionaldehyde	0.996	89 ± 2	97 ± 2	106 ± 5	1.9	1.7	4.5
3-(Trimethoxysilyl)propyl methacrylate	0.995	86 ± 6	84 ± 7	91 ± 4	7.5	8.5	4.6
4,4′-Difluorobenzophenone	0.995	96 ± 5	101 ± 2	102 ± 4	4.9	1.6	3.9
4-Methylphenol	0.992	97 ± 5	87 ± 9	94 ± 3	4.8	9.9	3.3
4-tert-Butylphenol	0.993	88 ± 9	90 ± 2	98 ± 4	10.1	2.0	3.9
Allyl methacrylate	0.995	104 ± 7	109 ± 2	100 ± 4	6.9	2.1	3.6
α-Methylstyrene	0.994	98 ± 6	114 ± 1	107 ± 4	6.2	1.3	3.6
α-Pinene	0.994	112 ± 5	117 ± 2	109 ± 4	4.1	1.6	3.5
Benzaldehyde	0.991	100 ± 8	117 ± 2	107 ± 6	7.6	1.8	5.1
Benzophenone	0.997	102 ± 1	96 ± 1	100 ± 4	1.0	1.2	3.6
Benzyl butyl phthalate	0.987	90 ± 1	84 ± 4	93 ± 5	1.5	4.7	5.3
Benzyl methacrylate	0.996	92 ± 4	95 ± 2	101 ± 4	3.9	1.8	4.3
β-Pinene	0.994	109 ± 8	115 ± 2	109 ± 4	7.3	1.7	3.6

Bis(2-ethylhexyl) adipate	0.983	88 ± 2	87 ± 4	90 ± 6	2.7	4.8	7.0
Bis(2-ethylhexyl) phthalate	0.997	97 ± 2	99 ± 7	102 ± 2	2.3	7.4	2.1
Bis(4-chlorophenyl) sulphone	0.996	86 ± 9	82 ± 9	93 ± 3	10.2	11.4	3.1
Butyl acrylate	0.989	92 ± 6	112 ± 2	106 ± 3	6.8	1.8	2.8
Butyl benzoate	0.995	104 ± 5	106 ± 2	104 ± 4	5.0	1.4	3.8
Butyl lactate	-	-	-	-	-	-	-
Butyl methacrylate	0.991	104 ± 5	114 ± 1	107 ± 4	4.4	0.9	3.4
Butylated hydroxytoluene	0.992	108 ± 4	89 ± 1	97 ± 5	3.2	1.4	4.8
Camphor	0.991	99 ± 5	110 ± 2	106 ± 4	4.9	1.4	3.8
Cyclohexyl methacrylate	0.996	100 ± 4	107 ± 2	106 ± 4	4.4	1.5	3.6
Diallyl phthalate	0.997	92 ± 4	108 ± 1	99 ± 5	4.3	1.2	4.8
Dibutyl adipate	0.991	94 ± 2	88 ± 4	89 ± 4	2.0	4.9	4.8
Dibutyl maleate	0.992	94 ± 2	83 ± 6	91 ± 5	2.4	6.9	5.4
Dibutyl phthalate	0.996	94 ± 3	83 ± 5	95 ± 5	3.5	6.2	5.3
Diethyl phthalate	0.999	98 ± 3	97 ± 5	101 ± 2	2.9	5.4	2.0
Diisobutyl phthalate	0.996	93 ± 2	86 ± 2	98 ± 4	2.4	2.1	4.4
Dimethyl isophthalate	0.996	85 ± 2	92 ± 1	98 ± 5	2.9	1.1	4.9
Dimethyl terephthalate	0.994	82 ± 3	89 ± 1	94 ± 4	4.1	1.4	4.8
Diphenyl phthalate	0.980	88 ± 8	107 ± 9	96 ± 9	9.1	8.4	9.4
Diphenyl sulphone	0.993	86 ± 5	89 ± 1	94 ± 4	5.9	1.1	4.8
Divinyl benzene	0.995	97 ± 4	105 ± 2	105 ± 4	3.9	1.6	3.5
Ethyl benzoate	0.996	104 ± 6	110 ± 1	107 ± 4	5.9	0.9	4.0
Ethylene glycol dimethacrylate	0.995	87 ± 4	90 ± 6	96 ± 5	5.1	6.2	5.6

Etocrylene	0.995	86 ± 1	87 ± 3	95 ± 6	1.2	3.8	6.3
Isobutyl acrylate	0.984	92 ± 6	117 ± 2	108 ± 4	6.6	1.3	3.3
Lauryl acrylate	0.992	95 ± 3	82 ± 5	97 ± 5	2.8	6.1	5.1
Methyl benzoate	0.987	95 ± 6	108 ± 3	108 ± 4	6.8	2.9	3.7
Methyl dihydrojasmonate	0.995	93 ± 5	86 ± 4	95 ± 5	5.8	5.1	5.2
Methyl salicylate	0.995	88 ± 8	86 ± 5	97 ± 5	9.4	5.4	5.1
Octocrylene	0.982	101 ± 1	86 ± 6	88 ± 7	1.1	6.6	7.7
Phenol	-	-	-	-	-	-	-
Phenyl methacrylate	0.994	92 ± 3	101 ± 2	105 ± 4	3.2	1.5	3.6
Propyl benzoate	0.990	95 ± 5	109 ± 1	106 ± 4	5.6	1.3	3.7
Styrene	0.991	100 ± 7	114 ± 2	107 ± 4	6.7	1.4	3.7
tert-Butyl methacrylate	0.986	98 ± 7	116 ± 1	107 ± 4	7.6	0.9	3.3
Triethoxyvinylsilane	0.990	96 ± 8	109 ± 1	104 ± 4	8.0	0.9	3.6
Triethyl citrate	-	-	-	-	-	-	-
Triethyl phosphite	0.993	88 ± 3	94 ± 1	91 ± 3	3.7	1.1	3.8
Trimethylolpropane trimethacrylate	0.994	86 ± 3	87 ± 3	90 ± 6	3.6	3.2	6.3
Tri-n-butyl acetyl citrate	-	-	-	-	-	-	-
Vinyl laurate	0.995	86 ± 1	82 ± 9	94 ± 5	1.6	10.6	4.8
Vinyltrimethoxysilane	0.981	87 ± 9	94 ± 6	95 ± 7	10.3	6.4	7.4

[a] Coefficient of determination (R^2) of the calibration curve in the range 40-400 μg L^{-1}, n=6.

[b] Relative recovery values (%) obtained in the analysis of Sample 4, expressed as the mean value of three replicates ± standard deviation.

[c] Precision expressed as relative standard deviation (RSD,%), n=5.

* Analytes with no data did not meet the validation criteria of Document No. SANTE/12682/2019 [38].
